# Cigarette smoking and the risk of primary Sjögren’s syndrome: a nested case control study

**DOI:** 10.1186/s13075-017-1255-7

**Published:** 2017-03-07

**Authors:** Peter Olsson, Carl Turesson, Thomas Mandl, Lennart Jacobsson, Elke Theander

**Affiliations:** 1 Department of Clinical sciences, Malmö, Rheumatology, Lund University, Malmö, Sweden; 2 Department of Rheumatology, Skåne University Hospital, Lund University, Inga Marie Nilssons gata 32, 20502 Malmö, Sweden; 3 Department of Rheumatology and Inflammation research, The Sahlgrenska Academy, University of Gothenburg, Gothenburg, Sweden

**Keywords:** Primary Sjögren’s syndrome, Epidemiology, Smoking, Socio-economy, Risk factor

## Abstract

**Background:**

Smoking is reported to affect the risk of a number of chronic disorders, including rheumatic diseases. Previous cross-sectional studies have shown a lower frequency of smoking in patients with primary Sjögren’s syndrome (pSS). The aim of this study was to investigate the impact of smoking and socioeconomic status on the risk of subsequent diagnosis of pSS in a nested case-control study.

**Method:**

Participants in two large population-based health surveys who were later diagnosed with pSS were identified through linkage with the Malmö Sjögren’s Syndrome Register. Matched controls were obtained from the health surveys.

**Results:**

Sixty-three patients with pSS with pre-diagnostic data from the health surveys were identified. Current smoking was associated with a significantly lower risk of later being diagnosed with pSS (odds ratio (OR) 0.3; 95% CI 0.1–0.6). Furthermore, former smoking was associated with an increased risk of subsequent pSS diagnosis (OR 4.0; 95% CI 1.8–8.8) compared to never smoking. Similar results were found in a sub-analysis of patients with reported symptom onset after inclusion in the health surveys. Socioeconomic status and levels of formal education had no significant impact on the risk of later being diagnosed with pSS.

**Conclusion:**

In this nested case-control study, current smoking was associated with a reduced risk of subsequent diagnosis of pSS. In addition, former smoking was associated with an increased risk. Whether this reflects a biological effect of cigarette smoking or other mechanisms should be further investigated in future studies.

## Background

Primary Sjögren’s syndrome (pSS) is an autoimmune disease primarily affecting the salivary and lacrimal glands, causing oral and ocular dryness. Other organs such as lungs, kidneys, nervous system, and skin may also be affected in the disease [1]. pSS is associated with B-cell activation, presence of various autoantibodies, systemic symptoms and an increased risk of non-Hodgkin lymphoma [2, 3]. The onset of the disease is often insidious and development of autoantibodies is seen years before onset of clinical disease [4, 5].

The estimated prevalence varies widely. When using the American European consensus group criteria (AECG criteria) [6] the prevalence of pSS is estimated to be between 11.3 and 236.1/100,000 in different European populations. Annual incidence estimates vary between 3.1 and 5.3/100000 [7].

The aetiopathogenesis of pSS is poorly understood but it entails genetic factors [8], hormonal factors [9] and possible viral infections [10], while environmental triggers are less well-studied. Smoking is a widely known risk factor for several malignant diseases [11, 12], but also for chronic inflammatory disorders such as rheumatoid arthritis (RA), systemic lupus erythematosus (SLE) and Crohn’s disease [13, 14]. Retrospective studies and studies with exposure measured before diagnosis [15–17] in RA indicate that smoking is particularly associated with seropositive RA. In some diseases, such as ulcerative colitis, Behcet’s disease and Parkinson’s disease, smoking seems to have a protective effect [18–20]. Only three studies have specifically addressed the association between smoking and pSS and in none of them has smoking been assessed prior to pSS diagnosis [21–23].

The closeness of cigarette smoke to mucosal membranes and exocrine glands, and the effect of the cigarette smoke on the immune system, makes the influence of cigarette smoking on the development of pSS interesting. However, when studying associations with smoking, social status has to be taken into consideration because it can correlate with both smoking and other exposures such as environmental toxins and dietary factors. Finding epidemiological risk factors for pSS might help us understand the underlying pathogenesis and thus guide further studies. As smoking affects the development of several diseases, including rheumatic disease, we hypothesized that smoking also might affect the development of pSS. To our knowledge, this is the first nested case-control study on the relationship between smoking and socioeconomic status and subsequent diagnosis of pSS.

## Method

### Aim

The aim of this study was to investigate the impact of smoking and socioeconomic status on the risk of later being diagnosed with pSS.

### Source populations

This nested case-control study used information from the Malmö Preventive Medicine Project (MPMP) and the Malmö Diet and Cancer Study (MDCS). The MPMP is a health survey performed in Malmö, Sweden (current population approximately 320,000, at the time of the survey 235,000) between 1974 and 1991 entailing 33,346 individuals. Of these, 22,444 were men born between 1921 and 1938 and 10,902 were women born between 1925 and 1938. The main purpose of the MPMP was to study risk factors for cardiovascular disease and alcohol abuse. Details on the recruitment have been described previously [24]. The overall participation rate was 71%.

The MDCS is a health survey performed in Malmö between 1991 and 1996 that included 30,447 individuals born between 1923 and 1945. The total source population was 74,138, and the participation rate was 41%. The survey has been described in detail previously [24, 25]. The main purpose of the MDCS was to study risk factors for cancer.

### Exposure information

Participants in the health surveys filled out self-administered questionnaires on personal and medical information, including smoking and level of education. After merging data from the MPMP and the MDCS, cases and controls were classified as current/not current smokers at the time of participation in the health survey, and also as current/former/never smokers. In the MDCS, formal education was classified as follows: ≤8 years of elementary school, 9–10 years, 11–12 years, >12 years and university degree. In the MPMP, information was available on whether the individual had attended elementary school, secondary school or higher education. A low level of formal education was classified in the MDCS as ≤8 years and in the MPMP as elementary school only.

Information on socioeconomic status had previously been incorporated in MPMP and MDCS by linking the databases with data on standardized socioeconomic status by occupation, based on information from Statistics Sweden, as previously described in detail [26]. The categorization of socioeconomic status by Statistics Sweden includes a division in groups corresponding to working class, middle class, and a group of self-employed inhabitants. For the present study, participants were classified as blue-collar workers or white-collar workers. Blue-collar workers included both skilled and unskilled manual workers, corresponding to the working-class group from Statistics Sweden. White-collar workers included non-manual employees of high level (occupations normally requiring six years of post-comprehensive school education, e.g. higher civil servants and executives), medium level (occupations normally requiring three years but not six years of post-comprehensive school education, with or without subordinates) and low level (normally requiring two but not three years of post-comprehensive school education with or without subordinates) and self-employed professionals such as architects and lawyers.

### Cases and controls

To identify pSS cases with pre-diagnostic information in the health surveys, the MPMP and the MDCS were linked to the Malmö Sjögren’s Syndrome Register (MSSR). The MSSR is a research database, in which consecutive patients with pSS have been included since 1984. At the time of the present study 409 patients were included in the cohort. Since 2002 only patients fulfilling the AECG criteria have been included. Patients included before 2002 have been assessed retrospectively and classified according to the AECG criteria. Only patients with a first pSS diagnosis after inclusion in the MPMP or MDCS, and who fulfilled the AECG criteria, were included in the present study.

The earliest available data were used for patients included in both surveys before diagnosis of pSS. Retrospective data on symptom onset obtained at the time of diagnosis were collected from the MSSR, thus were independently collected from the exposure data. Four controls for each case, matched for sex, age and time of inclusion, were obtained from the corresponding health survey. Controls were randomly selected from those who fulfilled the matching criteria using specially designed software. This nested case-control study design, based on the same source populations, has previously been used in several studies of predictors of RA [27–30] and giant cell arteritis [31].

### Statistics

Potential predictors of pSS were analyzed using conditional logistic regression (SPSS version 22). Each case and the corresponding controls were given a group number that was entered into the logistic regression models as a categorical variable. Analyses were also stratified by time to pSS diagnosis in the cases (above vs below the median). Separate analyses included only cases with documented onset of symptoms after inclusion in the health survey, and the corresponding controls.The two-sided Fisher exact test was used to analyze differences in immunologic markers after diagnosis of pSS by smoking status at inclusion in the health survey. A *p* value <0.05 was considered significant.

## Results

### Incident cases and controls

A total of 63 incident cases, patients who prior to a first diagnosis of pSS and fulfilment of the AECG criteria had been included either in the MPMP, the MDCS, or both, were identified. These are, from here, designated “pre-pSS patients”: 252 controls were obtained from the corresponding health surveys. Characteristics of the patients at diagnosis are shown in Table 1. The median time between inclusion in one of the health surveys and subsequent diagnosis was 8.2 years (IQR 2.4–14.1). Median age at inclusion in the health surveys was 51 years, and median age at diagnosis was 61 years. Demographics and information on exposures for cases and controls are listed in Table 2.

**Table 1:** Characteristics of incident cases of primary Sjögren’s syndrome

Characteristic	Patients (*n* = 63)
Sex (male/female) (*n*)	5/58
Age at diagnosis of pSS (years)	61 (54–69)
Time between inclusion and diagnosis (years)	8.2 (2.4–14.1)
Time between onset of symptoms and diagnosis (years)	4.0 (1.0–6.8)
Anti-SSA seropositive	59%
Anti-SSB seropositive	41%
ANA seropositive	73%
RF seropositive	57%
Lower lip salivary gland biopsy focus score ≥1	85%

Results are presented as numbers, median (IQR) or percentage in cases with available data. Missing data on time of symptom onset in 15 patients, Sjögren’s syndrome related antigen A, Ro (anti-SSA), Sjögren’s syndrome related antigen B, La (anti-SSB) and antinuclear antibodies (ANA) in 1 patient, rheumatoid factor (RF) in 2 patients and lower lip salivary gland biopsy focus score in 6 patients. *pSS* Primary Sjögren’s syndrome

**Table 2:** Demographic and exposure information in pre-primary Sjögren's syndrome cases and controls

	Cases (*n* = 63)	Controls (*n* = 252)	*P* value**
Female sex, *n* (%)	58/63 (92)	232/252 (92)	1.00
Age at inclusion in the population survey, mean (IQR), years	52.6 (49.0–56.0)	52.7 (49.0–56.0)	0.94
Current smoker, *n* (%^a^)	10/60 (17)	92/251 (37)	0.003
Former smoker, *n* (%^a^)	32/60 (53)	63/251 (25)	< 0.001
Never smoker, *n* (%^a^)	18/60 (30)	96/251 (38)	0.23
White-collar worker, *n* (%^a^)	26/54 (48)	131/236 (56)	0.33
Blue-collar worker, *n* (%^a^)	22/54 (41)	89/236 (38)	0.68
Other socioeconomic status, *n* (%^a^)	6/54 (11)	16/236 (7)	0.28
Low level of formal education, *n* (%^a^)	25/58 (43)	99/243 (41)	0.73
Medium/high level of formal education, *n* (%^a^)	33/58 (57)	144/243 (59)	0.73

^a^Percentage of individuals with available data. **Mann-Whitney *U* test

### Smoking and the risk of pSS

Information on smoking status was available in 60 cases. The distribution of current, former and never smokers differed between controls and pre-pSS patients with a higher proportion of former smokers among pre-pSS patients (Table 2). In conditional logistic regression analysis, current smoking at the time of inclusion in the health surveys was associated with a significantly lower risk of later being diagnosed with pSS compared to current non-smoking (OR 0.3; 95% CI 0.1–0.6). Former smoking was associated with a higher risk of being diagnosed with pSS, both compared to never smoking (OR 4.0; 95% CI 1.8–8.8) and current smoking (OR 7.8; 95% CI 3.1–19.9) (Table 3).

**Table 3:** Association between smoking status, socioeconomic status and level of formal education and development of primary Sjögren’s syndrome

		All pSS cases (*n* = 63) and matched controls (*n* = 252) OR (95% CI)	Exposed (cases/controls)	pSS cases with documented symptom onset after inclusion (*n* = 28) and matched controls (*n* = 112) OR (95% CI)	Exposed (cases/controls)
Smoking	Not current smokers	Reference	50/159	Reference	22/74
	Current smokers	0.3 (0.1–0.6)	10/92	0.2 (0.1–0.9)	4/38
	Never smokers	Reference	18/96	Reference	10/43
	Former smokers	4.0 (1.8–8.8)	32/63	1.7 (0.6–5.6)	12/31
	Current smokers	0.5 (0.2–1.3)	10/92	0.3 (0.1–1.5)	4/38
Socioeconomic status	White-collar worker	Reference	26/131	Reference	11/53
	Blue-collar worker	1.4 (0.7–2.8)	22/89	0.8 (0.2–2.5)	9/40
	Other	2.4 (0.7–8.4)	6/16	1.0 (0.3–3.2)	4/11
Education	Medium/high	Reference	33/144	Reference	11/55
	Low	1.1 (0.6–2.1)	25/99	1.4 (0.5–4.0)	13/49

Smoking status and level of formal education were assessed at inclusion in the health surveys. Patients were subsequently diagnosed with primary Sjögren’s syndrome (pSS) after a median of 8.2 years (IQR 2.4–14.1) after inclusion

### Sensitivity analyses

Retrospective data from the MSSR on symptom onset were available in 49 cases. There were 28 patients who had symptom onset >1 year after inclusion in the population surveys (range 2–24, median 6.5, IQR 3.25–14.75), and information on smoking status was available in 26 of these patients. In analyses restricted to these cases and their controls, current smoking was still associated with a lower risk of later being diagnosed with pSS compared with current non-smoking, and there was a trend towards an association between former smoking and subsequent pSS diagnosis (Table 3). In order to answer the question as to whether cessation of smoking was due to symptoms of pSS, we analyzed the time window between symptom onset and cessation of smoking. Among the cases with symptom onset >1 year after inclusion in the populations surveys, there were 12 patients who were former smokers. The time period between cessation of smoking and symptom onset was >5 years in all these cases.

In the whole group of pre-pSS patients who were former smokers and for whom information was available on symptom onset (n = 23), including those with symptom onset before inclusion in the health surveys, cessation of smoking occurred >5 years before symptom onset in 20 cases, 4 years before symptom onset in 1 case and after symptom onset in 1 case.

### Socioeconomic status, level of formal education and the risk of pSS

Socioeconomic status, comparing blue-collar workers and white-collar workers, did not affect the risk of being diagnosed with pSS. Furthermore, there was no association between the level of formal education and subsequent diagnosis of pSS (Table 3).

### Stratified analyses

To further examine the relationship between smoking or other exposures and pSS, analyses were performed separately in cases with a time period between inclusion in one of the health surveys and diagnosis of pSS that was above or below the median (8.2 years) and in the corresponding controls. The results in these subgroups were similar to those in the whole group (Table 4).

**Table 4:** Associations stratified by time from inclusion to diagnosis of primary Sjögren’s syndrome

		Time period ≤8.2 years (*n* = 32) OR (95% CI)	Exposed (cases/controls)	Time period >8.2 years (*n* = 31) OR (95% CI)	Exposed (cases/controls)
Smoking	Not current smokers	Reference	27/87	Reference	23/72
	Current smokers	0.3 (0.1–0.9)	4/40	0.3 (0.1–0.8)	6/52
	Never smokers	Reference	11/58	Reference	7/38
	Former smokers	4.9 (1.6–15.2)	16/29	3.4 (1.1–10.8)	16/34
	Current smokers	0.5 (0.1–1.8)	4/40	0.5 (0.1–2.0)	6/52
Socioeconomic status	White-collar worker	Reference	13/65	Reference	13/66
	Blue-collar worker	1.4 (0.5–3.8)	11/51	1.3 (0.5–3.7)	11/38
	Other	2.0 (0.3–14.3)	2/6	2.6 (0.5–14.4)	4/10
Education	Medium/high	Reference	18/74	Reference	15/70
	Low	1.1 (0.4–2.5)	13/53	1.1 (0.4–3.2)	12/46

Relationship between smoking, socioeconomic status and level of formal education stratified by time from inclusion in the health surveys to diagnosis of primary Sjögren’s syndrome

Anti-Sjögren’s syndrome related antigen A, Ro (anti-SSA) antibody positivity or presence or absence of focal sialadenitis at the time of diagnosis was not significantly different between individuals who were currently or not currently smoking at the time of inclusion in the health surveys, nor between those who had ever smoked or never smoked, or those who were currently smoking, were former smokers, or had never smoked (Table 5).

**Table 5:** Immunologic markers at primary Sjögren’s syndrome diagnosis or later by smoking status at inclusion *n* (% of available)

	Current smoker	*P* value*	Former smoker	*P* value*	Never smoker	*P* value*
Anti-SSApositive, *n* (%)	8 (80)	0.29	18 (56)	0.60	10 (56)	0.78
Anti- SSB positive, *n* (%)	7 (70)	0.07	11 (34)	0.43	6 (33)	0.57
ANA positive, *n* (%)	9 (90)	0.26	20 (63)	0.08	15 (83)	0.35
RF positive, *n* (%)	4 (44)	0.72	16 (50)	0.60	12 (67)	0.26
Autoantibody^a^ negative	1 (10)	1.00	7 (22)	0.16	1 (6)	0.26
Positive lower lip biopsy, *n* (%)	8 (80)	0.63	23 (82)	0.71	15 (94)	0.41

Smoking status at inclusion in the health surveys. Primary Sjögren’s syndrome was diagnosed at a median of 8.2 years (IQR 2.4–14.1) after inclusion. *Two-sided Fisher exact test. ^a^Patients who were negative for anti-Sjögren’s syndrome related antigen A, Ro (anti-SSA), anti-Sjögren’s syndrome related antigen B, La (anti-SSB), antinuclear antibody (ANA) and rheumatoid factor (RF)

## Discussion

In this study, being a current smoker was associated with a lower risk of later being diagnosed with pSS. Former smokers had a higher risk of subsequently being diagnosed with pSS, compared both to never smokers and current smokers. The same pattern is seen in ulcerative colitis [18]. In Parkinson’s disease, there is also an inverse association between cigarette smoking and development of the disease [20]. Interestingly, in three previous studies specifically addressing the association between smoking and pSS, there were fewer current smokers and more former smokers among pSS patients. The first study involved 355 pSS patients in whom there was a lower frequency of focal sialadenitis in lower lip biopsies and a lower frequency of anti-SSA positivity in patients with pSS who were smokers [21]. However, as the Copenhagen criteria were used, in which the presence of anti-SSA/SSB or presence of focal sialadenitis are not mandatory, several patients not fulfilling the AECG criteria were included in that study. One interpretation of that study is that within a population of individuals with sicca symptoms, smoking is negatively associated with markers of autoimmune sialadenitis.

The second study was a cross-sectional study of 140 patients, fulfilling the AECG criteria, in which there were also more former smokers and fewer current smokers among patients with pSS [22]. Finally, in a cross-sectional study of 207 pSS patients fulfilling the AECG criteria, there were fewer current smokers, but more former smokers compared to healthy controls [23]. The characteristics of the patients in that study were similar to the characteristics after diagnosis of the pSS patients in the present study, except that they had a higher frequency of positive lip biopsies (99.5% compared to 85% in the present study). In three other studies, which investigated cardiovascular risk factors in patients with pSS, there were fewer current smokers in one study [32], and in the other two studies there were fewer ever smokers [33, 34], compared to controls. In the latter two, there was no information on the numbers of current and former smokers.

Smoking is shown to cause a short-term increase in salivary flow rate (SRF) but a long-term reduced SFR [35, 36]. Cigarette smoking is also shown to cause ocular dryness when comparing chronic smokers with healthy controls [37]. A potential explanation for the smaller numbers of current smokers and for the greater numbers of former smokers in cross-sectional studies of pSS is that oral and ocular dryness and pulmonary symptoms make the patients more prone to quit smoking, both due to dryness and the irritation caused by the smoke. Interestingly, in this study, cessation of smoking in the majority of pSS patients who were former smokers did not occur in close temporal proximity to symptom onset and the impact of smoking was similar in cases with a longer and shorter time to diagnosis, supporting the possibility that smoke cessation of smoking might not be a consequence of sicca symptoms. Instead, it may influence the development or progression of the disease. Still, we cannot exclude that very early dryness, occurring before the reported onset of symptoms, could influence the cessation of smoking.

Low levels of formal education and a poor socioeconomic status have been shown to predict a number of chronic disorders [17, 26, 38, 39]. In contrast, in the present study, the level of education and socioeconomic status based on current occupation had no major impact on the risk of being diagnosed with pSS. This suggests that the observed negative association with smoking is probably not due to underlying exposures related to socioeconomic status, although we cannot exclude the possibility that other life style factors contribute to the development of pSS.

Cigarette smoke contains over four thousand different compounds [40]. Nicotine is known to interact in the immune system and nicotine receptors are present on macrophages, T cells and B cells [41–43]. Many other compounds in cigarette smoke can also affect the immune system [44]. Several studies have shown that cigarette smoke has effects on the immune system, which reasonably could affect the development of different diseases. As cigarette smoke, among other things, is shown to affect neutrophils, macrophages, T cells, B cells, dendritic cells, and cytokine production (reviewed in [40 − 42]), it is hard to predict exactly how cigarette smoke might affect the development of pSS [44–46]. One possible way is by inhibition of B cells: several studies have identified decreased levels of salivary IgA and decreased levels of serum IgG, and decreased levels of serum IgA and IgM and increased levels of IgE in smokers [46]. Furthermore, significant increases in IgM and IgG in subjects who stop smoking have been reported [47] and these changes seem to persist in former smokers for years after cessation of smoking [48].

pSS is characterized by B-cell activation with high serum IgG-levels and a high frequency of autoantibodies such as RF, anti-SSA and anti-SSB [1]. A reason why current smoking could be protective against pSS could be a suppressing effect of smoking on the B cells directly or by reduced production of B-cell activating factor [49, 50]. In predisposed individuals who stop smoking, taking away the inhibitory effect of cigarette smoke on the B cells could lead to activation of autoreactive B cells that might trigger the development of the disease. The association between smoking and anti-citrullinated peptide antibody (ACPA)-positive RA, on the other hand, most likely reflects other mechanisms specifically related to RA, such as increased protein citrullination leading to early development of ACPA [51].

As models of autoimmune diseases and the effect of cigarette smoking, inflammatory bowel diseases are interesting because smokers have a higher risk of Crohn’s disease but a lower risk of ulcerative colitis. Studies on ulcerative colitis and Crohn’s disease have identified distinct cytokine profiles, whereby Crohn’s disease is characterized by a T helper (Th)1 profile [52, 53], while in ulcerative colitis a Th2 cytokine profile has been observed [54, 55], with upregulated levels of IL-33 [56]. IL-33 is a nuclear cytokine of the IL-1 family, which is constitutively expressed in epithelial barrier tissues and plays an important role in Th2 immunity [57]. Recently, increased IL-33 in the salivary glands and in serum have been linked to pSS [58]. Interestingly, in a murine model, it has also been shown that exposure of cigarette smoke upregulates IL-33 but simultaneously changes the immune response of IL-33 from Th2 to Th1 response [59]. Hypothetically, this could explain why smoking may be protective against ulcerative colitis, and potentially also against pSS.

As the patients included in the MSSR are consecutive patients at the only department of rheumatology in the area, we consider the patients in the present study to be representative of the pSS population. Strengths of the present study include the study design, with exposure measured in a standardized manner before pSS diagnosis. The availability of two population-based surveys from a well-defined catchment area and the well-characterized patients with pSS in the MSSR are other important assets of the study.

Limitations of the study are mainly related to the relatively small number of cases. As the number of patients is small there is a risk that the patients are not representative of the whole pSS cohort. Unfortunately, quantitative information on smoking history (i.e. pack-years) was not available. Furthermore, smoking status was only assessed at one time point. All patients came from the same catchment area, which can influence the external validity of the study. With a disease like pSS, there is also always uncertainty about when the disease actually starts. However, in sub-analyses including only cases with symptom onset after the collection of survey data, we calculated similar point estimates on the association between smoking history and pSS, suggesting that reversed causality does not explain our findings.

## Conclusion

In conclusion, this study shows that current smoking was associated with a lower risk of later being diagnosed with pSS, whereas former smoking was associated with a higher risk. This pattern is similar to that seen in some other autoimmune diseases, e.g. ulcerative colitis and Parkinson’s disease, and may reflect immunomodulatory effects of smoking and cessation of smoking. These results from the first nested case-control study of smoking and pSS are consistent with previous observations from cross-sectional studies, and suggest that smoking might be protective against pSS. However, given the insidious onset of the disease, the limited number of patients included in the present study and the fact that smoking status was only assessed at one time point, the results of this study should be confirmed in subsequent prospective studies.

## Abbreviations

ACPA: Anti-citrullinated peptide antibody
AECG criteria: American European Consensus Group criteria
ANA: Antinuclear antibodies
BAFF: B-cell activating factor
CI: Confidence interval
IFN: Interferon
Ig: Immunoglobulin
IL: Interleukin
IQR: Interquartile range
MDCS: Malmö Diet and Cancer study
MPMP: Malmö Preventive Medicine Program
MSSR: Malmö Sjögren’s Syndrome Register
pSS: Primary Sjögren’s syndrome
RA: Rheumatoid arthritis
RF: Rheumatoid factor
SFR: Salivary flow rate
SLE: Systemic lupus erythematosus
SSA: Sjögren’s syndrome related antigen A, Ro
SSB: Sjögren’s syndrome related antigen B, La

## References

[1] Garcia-Carrasco M, Ramos-Casals M, Rosas J, Pallares L, Calvo-Alen J, Cervera R, Font J, Ingelmo M (2002). Primary Sjogren syndrome: clinical and immunologic disease patterns in a cohort of 400 patients. Medicine (Baltimore).

[2] Theander E, Henriksson G, Ljungberg O, Mandl T, Manthorpe R, Jacobsson LT (2006). Lymphoma and other malignancies in primary Sjogren’s syndrome: a cohort study on cancer incidence and lymphoma predictors. Ann Rheum Dis.

[3] Nishishinya MB, Pereda CA, Munoz-Fernandez S, Pego-Reigosa JM, Rua-Figueroa I, Andreu JL, Fernandez-Castro M, Rosas J, Loza Santamaria E (2015). Identification of lymphoma predictors in patients with primary Sjogren’s syndrome: a systematic literature review and meta-analysis. Rheumatol Int.

[4] Jonsson R, Theander E, Sjostrom B, Brokstad K, Henriksson G (2013). Autoantibodies present before symptom onset in primary Sjogren syndrome. JAMA.

[5] Theander E, Jonsson R, Sjostrom B, Brokstad K, Olsson P, Henriksson G (2015). Prediction of Sjogren’s syndrome years before diagnosis and identification of patients with early onset and severe disease course by autoantibody profiling. Arthritis Rheumatol.

[6] Vitali C, Bombardieri S, Jonsson R, Moutsopoulos HM, Alexander EL, Carsons SE, Daniels TE, Fox PC, Fox RI, Kassan SS (2002). Classification criteria for Sjogren’s syndrome: a revised version of the European criteria proposed by the American-European Consensus Group. Ann Rheum Dis.

[7] Qin B, Wang J, Yang Z, Yang M, Ma N, Huang F, Zhong R (2015). Epidemiology of primary Sjogren’s syndrome: a systematic review and meta-analysis. Ann Rheum Dis.

[8] Bolstad AI, Jonsson R (2002). Genetic aspects of Sjogren’s syndrome. Arthritis Res.

[9] Porola P, Virkki L, Przybyla BD, Laine M, Patterson TA, Pihakari A, Konttinen YT (2008). Androgen deficiency and defective intracrine processing of dehydroepiandrosterone in salivary glands in Sjogren’s syndrome. J Rheumatol.

[10] Croia C, Astorri E, Murray-Brown W, Willis A, Brokstad KA, Sutcliffe N, Piper K, Jonsson R, Tappuni AR, Pitzalis C (2014). Implication of Epstein-Barr virus infection in disease-specific autoreactive B cell activation in ectopic lymphoid structures of Sjogren’s syndrome. Arthritis Rheumatol.

[11] Doll R, Hill AB (1956). Lung cancer and other causes of death in relation to smoking; a second report on the mortality of British doctors. Br Med J.

[12] Morgan RW, Jain MG (1974). Bladder cancer: smoking, beverages and artificial sweeteners. Can Med Assoc J.

[13] Sugiyama D, Nishimura K, Tamaki K, Tsuji G, Nakazawa T, Morinobu A, Kumagai S (2010). Impact of smoking as a risk factor for developing rheumatoid arthritis: a meta-analysis of observational studies. Ann Rheum Dis.

[14] Cosnes J (2004). Tobacco and IBD: relevance in the understanding of disease mechanisms and clinical practice. Best Pract Res Clin Gastroenterology.

[15] Kallberg H, Ding B, Padyukov L, Bengtsson C, Ronnelid J, Klareskog L, Alfredsson L, Group ES (2011). Smoking is a major preventable risk factor for rheumatoid arthritis: estimations of risks after various exposures to cigarette smoke. Ann Rheum Dis.

[16] Costenbader KH, Feskanich D, Mandl LA, Karlson EW (2006). Smoking intensity, duration, and cessation, and the risk of rheumatoid arthritis in women. Am J Med.

[17] Bergstrom U, Jacobsson LT, Nilsson JA, Wirfalt E, Turesson C (2013). Smoking, low formal level of education, alcohol consumption, and the risk of rheumatoid arthritis. Scand J Rheumatol.

[18] Calkins BM (1989). A meta-analysis of the role of smoking in inflammatory bowel disease. Dig Dis Sci.

[19] Rizvi SW, McGrath H (2001). The therapeutic effect of cigarette smoking on oral/genital aphthosis and other manifestations of Behcet’s disease. Clin Exp Rheumatol.

[20] van der Mark M, Nijssen PC, Vlaanderen J, Huss A, Mulleners WM, Sas AM, van Laar T, Kromhout H, Vermeulen R (2014). A case-control study of the protective effect of alcohol, coffee, and cigarette consumption on Parkinson disease risk: time-since-cessation modifies the effect of tobacco smoking. PLoS One.

[21] Manthorpe R, Benoni C, Jacobsson L, Kirtava Z, Larsson A, Liedholm R, Nyhagen C, Tabery H, Theander E (2000). Lower frequency of focal lip sialadenitis (focus score) in smoking patients. Can tobacco diminish the salivary gland involvement as judged by histological examination and anti-SSA/Ro and anti-SSB/La antibodies in Sjogren’s syndrome?. Ann Rheum Dis.

[22] Priori R, Medda E, Conti F, Cassara EA, Sabbadini MG, Antonioli CM, Gerli R, Danieli MG, Giacomelli R, Pietrogrande M (2007). Risk factors for Sjogren’s syndrome: a case-control study. Clin Exp Rheumatol.

[23] Karabulut G, Kitapcioglu G, Inal V, Kalfa M, Yargucu F, Keser G, Emmungil H, Gokmen NM, Kocanaogullari H, Aksu K (2011). Cigarette smoking in primary Sjogren’s syndrome: positive association only with ANA positivity. Mod Rheumatol.

[24] Nilsson PM, Nilsson JA, Berglund G (2004). Family burden of cardiovascular mortality: risk implications for offspring in a national register linkage study based upon the Malmo Preventive Project. J Intern Med.

[25] Manjer J, Carlsson S, Elmstahl S, Gullberg B, Janzon L, Lindstrom M, Mattisson I, Berglund G (2001). The Malmo Diet and Cancer Study: representativity, cancer incidence and mortality in participants and non-participants. Eur J Cancer Prev.

[26] Bergstrom U, Jacobsson LT, Nilsson JA, Berglund G, Turesson C (2011). Pulmonary dysfunction, smoking, socioeconomic status and the risk of developing rheumatoid arthritis. Rheumatology (Oxford).

[27] Pikwer M, Bergstrom U, Nilsson JA, Jacobsson L, Berglund G, Turesson C (2009). Breast feeding, but not use of oral contraceptives, is associated with a reduced risk of rheumatoid arthritis. Ann Rheum Dis.

[28] Pikwer M, Giwercman A, Bergstrom U, Nilsson JA, Jacobsson LT, Turesson C (2014). Association between testosterone levels and risk of future rheumatoid arthritis in men: a population-based case-control study. Ann Rheum Dis.

[29] Turesson C, Bergstrom U, Pikwer M, Nilsson JA, Jacobsson LT (2016). A high body mass index is associated with reduced risk of rheumatoid arthritis in men, but not in women. Rheumatology (Oxford).

[30] Turesson C, Bergstrom U, Pikwer M, Nilsson JA, Jacobsson LT (2015). High serum cholesterol predicts rheumatoid arthritis in women, but not in men: a prospective study. Arthritis Res Ther.

[31] Jakobsson K, Jacobsson L, Warrington K, Matteson EL, Liang K, Melander O, Turesson C (2015). Body mass index and the risk of giant cell arteritis: results from a prospective study. Rheumatology (Oxford).

[32] Juarez M, Toms TE, de Pablo P, Mitchell S, Bowman S, Nightingale P, Price EJ, Griffiths B, Hunter J, Gupta M (2014). Cardiovascular risk factors in women with primary Sjogren’s syndrome: United Kingdom primary Sjogren’s syndrome registry results. Arthritis Care Res (Hoboken).

[33] Perez-De-Lis M, Akasbi M, Siso A, Diez-Cascon P, Brito-Zeron P, Diaz-Lagares C, Ortiz J, Perez-Alvarez R, Ramos-Casals M, Coca A (2010). Cardiovascular risk factors in primary Sjogren’s syndrome: a case-control study in 624 patients. Lupus.

[34] Bartoloni E, Baldini C, Schillaci G, Quartuccio L, Priori R, Carubbi F, Bini V, Alunno A, Bombardieri S, De Vita S (2015). Cardiovascular disease risk burden in primary Sjogren’s syndrome: results of a population-based multicentre cohort study. J Intern Med.

[35] Khan GJ, Javed M, Ishaq M (2010). Effect of smoking on salivary flow rate. Gomal J Med Sci.

[36] Rad M, Kakoie S, Niliye Brojeni F, Pourdamghan N (2010). Effect of Long-term Smoking on Whole-mouth Salivary Flow Rate and Oral Health. J Dent Res Dent Clin Dent Prospects.

[37] Sayin N, Kara N, Pekel G, Altinkaynak H (2014). Effects of chronic smoking on central corneal thickness, endothelial cell, and dry eye parameters. Cutan Ocul Toxicol.

[38] Pincus T, Callahan LF, Burkhauser RV (1987). Most chronic diseases are reported more frequently by individuals with fewer than 12 years of formal education in the age 18-64 United States population. J Chronic Dis.

[39] Li X, Sundquist J, Sundquist K (2008). Socioeconomic and occupational risk factors for rheumatoid arthritis: a nationwide study based on hospitalizations in Sweden. J Rheumatol.

[40] Smith CJ, Hansch C (2000). The relative toxicity of compounds in mainstream cigarette smoke condensate. Food Chem Toxicol.

[41] Wang H, Yu M, Ochani M, Amella CA, Tanovic M, Susarla S, Li JH, Wang H, Yang H, Ulloa L (2003). Nicotinic acetylcholine receptor alpha7 subunit is an essential regulator of inflammation. Nature.

[42] Nizri E, Irony-Tur-Sinai M, Lory O, Orr-Urtreger A, Lavi E, Brenner T (2009). Activation of the cholinergic anti-inflammatory system by nicotine attenuates neuroinflammation via suppression of Th1 and Th17 responses. J Immunol.

[43] Skok MV, Grailhe R, Agenes F, Changeux JP (2007). The role of nicotinic receptors in B-lymphocyte development and activation. Life Sci.

[44] Sopori M (2002). Effects of cigarette smoke on the immune system. Nat Rev Immunol.

[45] Arnson Y, Shoenfeld Y, Amital H (2010). Effects of tobacco smoke on immunity, inflammation and autoimmunity. J Autoimmun.

[46] Edwards D (2009). Immunological effects of tobacco smoking in “healthy” smokers. COPD.

[47] Hersey P, Prendergast D, Edwards A (1983). Effects of cigarette smoking on the immune system. Follow-up studies in normal subjects after cessation of smoking. Med J Aust.

[48] Mili F, Flanders WD, Boring JR, Annest JL, Destefano F (1991). The associations of race, cigarette smoking, and smoking cessation to measures of the immune system in middle-aged men. Clin Immunol Immunopathol.

[49] Fusby JS, Kassmeier MD, Palmer VL, Perry GA, Anderson DK, Hackfort BT, Alvarez GK, Cullen DM, Akhter MP, Swanson PC (2010). Cigarette smoke-induced effects on bone marrow B-cell subsets and CD4+:CD8+ T-cell ratios are reversed by smoking cessation: influence of bone mass on immune cell response to and recovery from smoke exposure. Inhal Toxicol.

[50] Wang J, Li Q, Xie J, Xu Y (2015). Cigarette smoke inhibits BAFF expression and mucosal immunoglobulin A responses in the lung during influenza virus infection. Respir Res.

[51] Makrygiannakis D, Hermansson M, Ulfgren AK, Nicholas AP, Zendman AJ, Eklund A, Grunewald J, Skold CM, Klareskog L, Catrina AI (2008). Smoking increases peptidylarginine deiminase 2 enzyme expression in human lungs and increases citrullination in BAL cells. Ann Rheum Dis.

[52] Parronchi P, Romagnani P, Annunziato F, Sampognaro S, Becchio A, Giannarini L, Maggi E, Pupilli C, Tonelli F, Romagnani S (1997). Type 1 T-helper cell predominance and interleukin-12 expression in the gut of patients with Crohn’s disease. Am J Pathol.

[53] Sartor RB (2006). Mechanisms of disease: pathogenesis of Crohn’s disease and ulcerative colitis. Nat Clin Pract Gastroenterol Hepatol.

[54] Fuss IJ, Neurath M, Boirivant M, Klein JS, de la Motte C, Strong SA, Fiocchi C, Strober W (1996). Disparate CD4+ lamina propria (LP) lymphokine secretion profiles in inflammatory bowel disease. Crohn’s disease LP cells manifest increased secretion of IFN-gamma, whereas ulcerative colitis LP cells manifest increased secretion of IL-5. J Immunol.

[55] Heller F, Florian P, Bojarski C, Richter J, Christ M, Hillenbrand B, Mankertz J, Gitter AH, Burgel N, Fromm M (2005). Interleukin-13 is the key effector Th2 cytokine in ulcerative colitis that affects epithelial tight junctions, apoptosis, and cell restitution. Gastroenterology.

[56] Kobori A, Yagi Y, Imaeda H, Ban H, Bamba S, Tsujikawa T, Saito Y, Fujiyama Y, Andoh A (2010). Interleukin-33 expression is specifically enhanced in inflamed mucosa of ulcerative colitis. J Gastroenterol.

[57] Miller AM (2011). Role of IL-33 in inflammation and disease. J Inflamm (Lond).

[58] Awada A, Nicaise C, Ena S, Schandene L, Rasschaert J, Popescu I, Gangji V, Soyfoo MS (2014). Potential involvement of the IL-33-ST2 axis in the pathogenesis of primary Sjogren’s syndrome. Ann Rheum Dis.

[59] Kearley J, Silver JS, Sanden C, Liu Z, Berlin AA, White N, Mori M, Pham TH, Ward CK, Criner GJ (2015). Cigarette smoke silences innate lymphoid cell function and facilitates an exacerbated type I interleukin-33-dependent response to infection. Immunity.

